# Comparison of convective heat transfer for Kagome and tetrahedral truss-cored lattice sandwich panels

**DOI:** 10.1038/s41598-019-39704-2

**Published:** 2019-03-06

**Authors:** Guangmeng Yang, Chi Hou, Meiying Zhao, Wei Mao

**Affiliations:** 1 School of Aeronautics, Northwestern Polytechnical University, Xi’an, 710072 China; 2 Science and technology on space physics laboratory, Beijing, 100076 China

## Abstract

The aim of this paper is to make a thorough comparison between Kagome and tetrahedral truss-cored lattices both experimentally and numerically. Two titanium sandwich panels –one cored with the Kagome lattice and the other with the tetrahedral lattice –are manufactured by 3D printing technology. Comparisons of the thermal insulation, the inner flow pattern and the heat transfer between the two sandwich panels are completed subsequently according to the results from forced convective experiments and numerical simulation. Within the Reynolds number range of interest for this study, the Kagome lattice exhibits excellent heat dissipation compared with the tetrahedral lattice. In particular, when the cooling air flows in the direction OB of the two sandwich panels, the Kagome lattice provides an 8~37% higher overall Nusselt number for the sandwich panel compared to the tetrahedral lattice. The superiority of the Kagome lattice comes from a unique configuration in which the centre vertex acting as the vortex generator not only disturbs the primary flow but also induces more serious flow stagnation and separation. The complex fluid flow behaviours enhance heat transfer on both the endwalls and the trusses while causing a pressure drop that is almost two times higher than that of the tetrahedral lattice in the flow direction OB.

## Introduction

Lightweight sandwich panels cored with periodic cellular materials (PCMs) are promising structures for multifunctional applications in which thermal protection and load-bearing functions are required simultaneously, such as the wing leading edge of hypersonic vehicles, the combustion chamber of rockets, and the nose cone of re-entry vehicles^1–4^. Mechanically, for a given porosity, PCMs exhibit excellent compressive strength compared to metal foams and prismatic corrugations, and they even appear superior at relatively low densities^5,6^. Furthermore, their high specific surface areas endow them with comparable heat transfer performance as well as lower pressure drops to metal foam^1^. Additionally, the periodic geometric topologies of PCMs are facilitated through fabrication by using various manufacturing technologies, such as laser-cut-out lattice^7^, metal wire weaving^8,9^, metal sheet forming^10,11^, and investment casting^12^, which are well documented in the literature^1^. In view of these advantages, considerable efforts have been devoted to developing cost-effective and design-friendly PCMs, which mainly include woven textiles^13–15^, tetrahedral lattice^16^, pyramidal lattice^17–19^, X-type lattice^3,20,21^ and Kagome^22^.

Amongst the various PCMs, the truss-cored tetrahedral lattice has gained wide attention from many researchers due to its relatively simple geometric topology. Kim *et al*.^23–25^ thoroughly presented experimental and numerical studies of metallic tetrahedral unit cells with respect to the overall pressure drop and fluid flow characteristics. Their research revealed that tetrahedral lattice-frame materials (LFMs) induced horseshoe vortices and arch-shaped vortices, which led to almost seven times higher Nusselt number than that of a plain channel. Additionally, orientation effects were also considered: it was concluded that the thermal performance was similar, while the pressure drop depended strongly on the airflow orientation. Gao^26,27^ introduced a composite tetrahedral LFM and revealed the heat transfer mechanism by analysing the local thermal and fluid flow patterns. For a given porosity, Zhang^28^ compared the thermal performance between square and circular cross-section tetrahedral lattice cores and determined that a 13% to 16% higher Nusselt number was achieved for square ligaments compared with circular ligaments, which was due to the complex flow mixing. In addition, the effect of porosity was studied by Karen^4^, and the results showed that the high Nusselt number increased as porosity decreased due to the increased surface area and flow mixing.

Recently, a novel Kagome lattice morphologically formed by two tetrahedrons symmetric about the shared node has been regarded as one of the best truss-type lattice structures. For a fixed relative density, Hyun^29^ and Wang^12^ demonstrated that the Kagome exhibited better resistance to plastic bulking over tetrahedral and octet trusses. Further, Yan^3^ numerically compared the heat transfer characteristics of Kagome with those of other PCMs and revealed that Kagome provided up to 38% higher overall Nusselt numbers compared to tetrahedral lattices, which is comparable to X-type lattice. In view of the excellent thermo-mechanical properties of Kagome lattice, Hoffmann^22^ experimentally investigated the thermo-fluidic characteristics of Kagome metallic lattice, and the results showed that the topological orientation had an evident effect on both pressure drop and heat transfer. A similar conclusion was reached by Joo *et al*.^30,31^ in their research on a geometric-anisotropy Kagome truss-like PCM [called wire-woven bulk Kagome (WBK)]. They found that the heat transfer performance in most closed open orientations was consistently higher than that in most open orientations, which mainly contributed to the difference in the open area ratios of the two orientations. The thermal performance of WBK was investigated experimentally by Feng^32^, and it was found that the WBK can compete favourably with the current best cellular heat dissipation media under forced-air convection. However, a systematic comparison of the heat transfer between truss-cored Kagome and WBK was conducted by Shen and Yan^33^. They revealed that the truss-cored Kagome exhibits a 26~31% higher overall Nusselt number compared to WBK with a similar pressure drop, indicating that the geometrical topology was significant in inducing a strong vortex flow for overall thermal performance improvement.

As mentioned above, the current studies on the geometric-anisotropy tetrahedral and Kagome lattices mainly focus on heat transfer performance and pressure drop by experimental measurement. Despite existing a degree of exploration into the flow feature, the insightful thermo-fluidic mechanisms that would elucidate the heat dissipation superiority of Kagome lattice to tetrahedral lattice as mentioned in^3^, are absent. Essentially, the heat transfer performance is dominated by heat exchange between the sandwich panel and the cooling medium, which is induced by the fluid flow behaviours. Thus, it is necessary to elaborate the overall and local flow features deeply to explain why the two lattice structures exhibit the difference in heat transfer performance. Further, another issue that has not been addressed in any of the reviewed literature is that compared to the tetrahedral lattice core, the compacted morphology of Kagome lattice core contributes to arranging more unit cells under the identical space dimension. Since in actual thermal protection structures, such as the combustion of a rocket, how to achieve the maximum thermal efficiency in a limited space dimension is particularly important. In this situation, the heat transfer performance for the two lattices deserves research.

To overcome these puzzles, titanium sandwich panels with truss-cored Kagome lattice and tetrahedral lattice are manufactured by 3D printing technique^34,35^ under the identical space dimension. The convective heat transfer experiments are carried out for the two lattices, and the air used as the cooling medium flows through the specimens in two perpendicular directions to take the topological orientation into consideration. Numerical simulation of the convective heat transfer process is performed subsequently to make a comparison of the overall heat transfer for the two panels. Particular focus is placed on revealing the distinctive local heat transfer characteristics and their contribution to overall heat transfer and hydraulic performance.

## Experimental Investigation

### Specimen details

**Figure 1:**
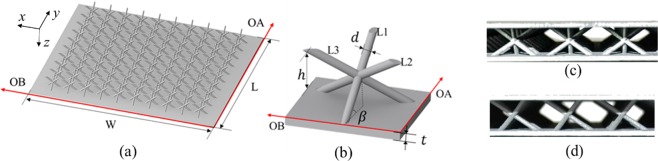
Test sample for Kagome lattice: (**a**) details of sandwich panel; (**b**) unit cell; (**c**) front view of unit cell along y-axis (OA); (**d**) front view of unit cell along x-axis (OB).

**Figure 2:**
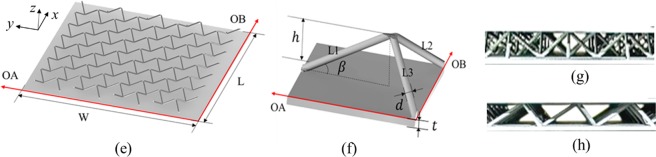
Test sample for tetrahedral lattice: (**a**) details of sandwich panel; (**b**) unit cell; (**c**) front view of unit cell along y-axis (OA); (**d**) front view of unit cell along x-axis (OB).

The unit cells of the two lattices in present study are designed in touch with each other to achieve the excellent thermal performance. The detailed illustration for the sandwich panels cored with Kagome and tetrahedral lattices are shown in Fig. 1 and Fig. 2, respectively. The corresponding unit cell and front views in two perpendicular orientations OA and OB for the two lattices are depicted in Fig. 1(b–d) and Fig. 2(b–d). Morphologically, Kagome trusses are intersected by the centre vertex in a unit cell, while tetrahedral trusses are connected to the facesheet of sandwich panel; therefore, the former facilitates a compacted arrangement of unit cells than the latter. The Kagome core shown in Fig. 1(a) contains ten unit cells along both the orientations OA and OB, and the tetrahedral core in Fig. 2(a) includes eight unit cells along the orientation OA and seven unit cells along the orientation OB with an arrangement in a staggered fashion. On the other hand, the sizes of the sandwich panel along the two perpendicular orientations are large enough to eliminate the sidewall effects on the experimental results^23^. The specimens are made of titanium alloy TC4 with a thermal conductivity of 8.79 W/(mK), and their geometric parameters are summarized in Table 1. The two sandwich panels have identical geometric sizes, except for the height because of manufacturing tolerance. The relative density for the Kagome lattice and tetrahedral lattice are calculated as 0.018 and 0.011, respectively, with the existence of a slight deviation of 0.007. 3D printing technology is used to fabricate the lattice core and panels simultaneously without the requirement for an assembly process. The integrative manufacturing method avoids the high thermal contact resistance between the sandwich panel and the lattice core, which can cause a significant knockdown in the heat transfer performance^36^.

**Table 1:** Geometrical parameters of the Kagome and tetrahedral lattice sandwich panels.

_Lattice_	h (mm)	d (mm)	t (mm)	W (mm)	L (mm)	β (deg)
Kagome	13.25	2.0	2.0	317.0	317.0	29.14
Tetrahedron	12.85	2.0	2.0	317.0	317.0	29.14

### Experimental apparatus and procedure

**Figure 3:**
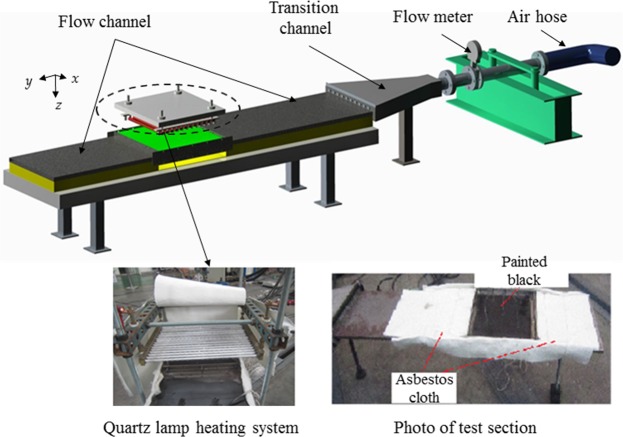
Schematic of experimental setup.

The experimental apparatus for forced air convection are illustrated in Fig. 3, which are configured with an air supply system, a test section and a data acquisition system.

The air supply system consists of an air hose, a transition channel and two flow channels. Compressed air from the air tank is supplied into the entry section of the circle air hose. Due to the dimensional difference between the air hose section and the specimen section, a transition channel is adopted to connect the air hose and the rectangular upstream flow channel so that the airflow can gradually transform into the stable laminar state before entering into the specimen. Another flow channel is placed downstream of the test section to ensure that the flow leaves the specimen steadily. Both of the upstream and downstream flow channels have the same length of 790 mm.

The test section contains the specimen and a quartz lamp heating system. A uniform heat flux is applied on the outer surface of the bottom substrate (named the heating surface) by the quartz lamp composed of etched Inconel heating pipes sandwiched between two Kapton films. The applied heat flux is controlled by an automatic system so that the temperature of the heating surface can be held at any specified value. The heating surface is painted black to better absorb the radiant energy from the quartz lamp. Finally, the test channel is sealed by thermal baffles and subsequently insulated by asbestos cloth.

**Figure 4:**
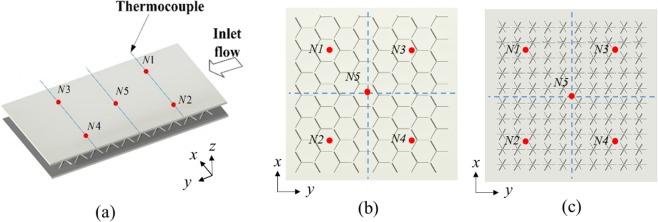
Position of thermocouples N1–N5 on cooling surface for specimen; (**a**) Schematic overview; (**b**) tetrahedral lattice; (**c**) Kagome lattice.

The measured data includes volume flow rates and temperature. A flow meter is installed at the entry section to obtain the inlet mass flow rate by using mass weight method. Ten thermocouples, divided into two groups (N1-N5 and N6-N10), are fixed on the heating surface and the outer surface of the upper substrate (named the cooling surface) to measure the facesheet temperature. Among them, the position of thermocouples N1-N5 for the two lattices is schematically illustrated in Fig. 4(a–c). Four thermocouples are correspondingly located at the centre point of the four uniform subdomains, while thermocouple N5 is installed at the centre of the cooling surface. Finally, the test status is considered to reach stabilization once the temperature measured by the thermocouple at the centre of the heating surface is within a 0.2 °C range in 3 min.

An uncertainty analysis is performed using the root mean square method described in Coleman and Steele^37^. The uncertainty of temperature from the thermocouple and is 0.1 °C, and the precision of flow rate from the flow meter is 0.5%. The density and viscosity of air are derived based on inlet static pressure and temperature, whose uncertainties are neglected. Therefore, the uncertainty for the Reynolds number is estimated as 0.5%.

### Experimental results

To investigate the effect of geometric morphology and topological orientation on thermal performance, the experiments are carried out in forced air convection along two perpendicular orientations, OA and OB, one for each of the two lattices. Thus, there are four test cases in total, labelled “Kagome OA”, “Kagome OB”, “Tetrahedron OA” and “Tetrahedron OB”. Three different flow velocities (5.0 m/s, 10.0 m/s and 15.0 m/s) are chosen to evaluate the heat transfer performance of the sandwich panels. The heating temperature, 300 °C, is measured by the average temperature of thermocouples N6–N10, expressed as:

1 $${T}_{h}=\sum _{n=6}^{10}{T}_{i}$$

Table 2 presents the measured flow velocity at the inlet section and the temperature distribution on the cooling surface. Although the actual flow velocity and the heating temperature are slightly different from the designed value for the four test cases, some certain conclusions can be made. Under an identical temperature load, the thermal insulation performance can be evaluated in terms of the mean temperature on the cooling surface. It can be seen that among the test cases, the Kagome OB exhibits the lowest mean temperature on the cooling surface in the entire range of the flow velocity, indicating that the Kagome OB has the best thermal insulation under the identical heating temperature. It is significant in engineering equipment that excellent thermal insulation performance is facilitated to provide the inner devices, such as electronic devices and working facilities, with a suitable working condition.

**Table 2:** Forc e convective experimental results for test samples.

Test case	Flow velocity m/s	Heating temperature °C	Bottom temperature °C					Mean temperature °C
			N1	N2	N3	N4	N5	
Kagome OA	6.8	299.9	57.2	54.4	104.9	85.8	82.4	76.94
	9.7	300.9	52.9	49.0	95.4	81.4	75.2	70.78
	15.2	303.6	47.3	43.8	83.7	72.8	64.3	62.38
Kagome OB	5.5	294.1	82.6	50.5	88.2	51.0	73.8	69.22
	9.4	297.0	64.2	41.4	71.5	41.5	59.9	55.70
	15.2	301.2	56.0	37.8	62.9	38.0	54.1	49.76
Tetrahedron OA	7.0	298.1	60.9	58.0	85.8	90.3	78.2	74.64
	10.5	297.7	56.2	52.8	76.5	81.1	70.9	67.50
	14.7	295.4	52.8	48.9	69.6	74.1	65.6	62.20
Tetrahedron OB	6.5	298.4	88.4	65.4	85.5	64.6	82.8	77.34
	11.0	298.3	71.6	51.2	66.3	49.5	65.4	60.80
	15.2	305.1	66.3	48.2	62.0	46.9	60.9	56.86

In addition, the measured temperature markedly decreases with the increment of the flow velocity because of the enhanced heat exchange between the lattice structure and the cooling air. Furthermore, note that for the thermocouples that are the same distance from the inlet section, the corresponding temperature should be similar in theory. Thus, it can be seen that within a test case, the temperature uniformity is excellent near the inlet section with a maximum deviation of 3.9 °C, while the phenomenon gets worse near the outlet section because of the developed thermal flow. For the four test cases, the tetrahedral lattice exhibits better temperature uniformity than the Kagome lattice, which may be attributed to the more complex flow mixing in the Kagome lattice. Hence, it is necessary to perform numerical analysis to investigate the detailed thermo-fluidic features for the two lattices in the next section.

## Numerical Simulation

### Numerical model

To explore the physical enhancement mechanism associated with the morphologies of the lattice structures, numerical simulation for convective heat transfer is carried out with the commercial software ANSYS 17.0^TM^.

**Figure 5:**
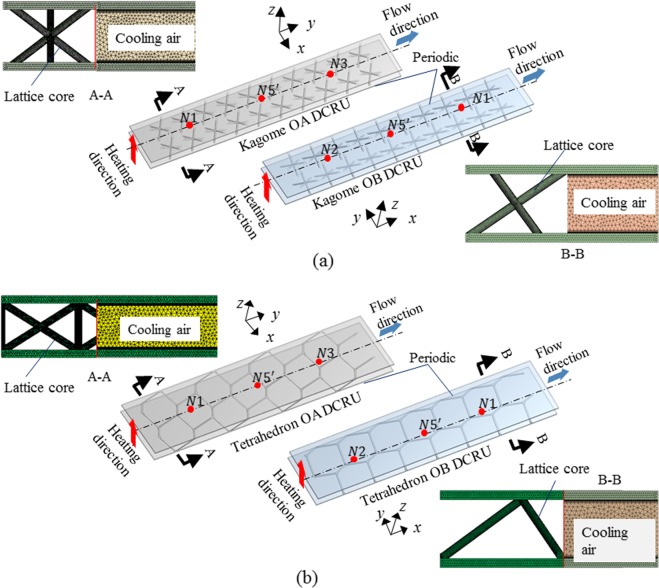
The double-channel representative units (DCRU) models: (**a**) Kagome OA and Kagome OB; (**b**) tetrahedron OA and tetrahedron OB.

The Kagome and tetrahedral lattice sandwich panels are modeled first in CATIA^TM^. Due to the geometrical periodicity of the lattice core, the typical double-channel representative unit (DCRU) comprised of two arrays of cells along the streamwise direction is adopted to reduce complexity and improve calculation efficiency. To draw a comparison with the temperature measured in experiments, the DCRU for case OA contains the thermocouples N1 and N3, and the DCRU for case OB includes the thermocouples N1 and N2. Additionally, the centre point of the DCRU is selected and marked as N5′ to compare with the thermocouple N5 as they are the same distance from the inlet section. The DCRU for Kagome and tetrahedral lattices along both the orientations OA and OB are presented in Fig. 5.

A hybrid mesh incorporating tetrahedral and prism grids is adopted for the complex fluid region by ICEM CFD 17.0, as illustrated in Fig. 5. For the flow boundary layers, prism elements with twenty layers are generated near the interface of the solid domain and the fluid domain in order to capture the detailed flow characteristics, while a coarse tetrahedral mesh is applied in the other regions. High mesh densities with approximately 3.0 × 10^7^ and 2.7 × 10^7^ cells are distributed in Kagome and tetrahedral numerical models, respectively, to eliminate the influence of mesh dependency.

The flow velocity, measured experimentally with a static temperature of 300 K, is defined as the inlet boundary condition. With consideration of robustness and numerical stability, the average static pressure is adopted as the outlet boundary condition. For the fluid-solid coupling interface, a conservative interface flux condition is adopted for heat transfer by incorporating a general grid interface (GGI) mesh connection. Translational periodicity boundary condition with zero pressure drop is used on the periodic surfaces for both the solid domain and air domain^3,28^, with the other surfaces of the computational domain set to be adiabatic. Considering that the heat flux applied on the heating surface is not measured directly in the force convective experiment, its value is derived based on the measured heating temperature. First, the initial heat flux is obtained by applying the average heating temperature in the numerical model. Then, the heat flux is adjusted and determined until the average heating temperature on the heating surface is identical to the experimental value. The method can eliminate the heat loss effectively during thermal radiation from the quartz lamp heating system to the specimen in experiment.

### Validation of numerical model

The numerical model is validated by comparing the calculated temperature of the thermocouples with the experimental values. The problem of incompressible steady-state flow and conjugated heat transfer is solved using the double precision solver ANASYS CFX 17.0 based on the finite volume method. For the turbulent flow condition, the shear stress transport (SST) model is employed due to its improving capability in predicting large flow separation, which has been widely adopted by Yan^3,20^ and Shen *et al*.^33^. Finally, the high resolution scheme with a second-order turbulence numeric is applied to discretize the momentum and energy equations. The solution is thought to be converged when the normalized residuals of all the governing terms are less than 10^−5^.

**Figure 6:**
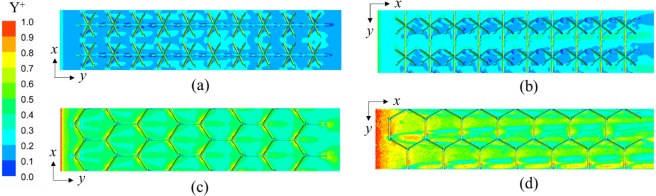
Dimensionless distance for the interfaces at the Reynolds number of 13000: (**a**) Kagome OA; (**b**) Kagome OB; (**c**) Tetrahedron OA; (**d**) Tetrahedron OB.

To validate the grid sensitivity, Fig. 6 first presents the yield of the dimensionless distance (y^+^) for four test cases at the highest Reynolds number of 13,000. It can be seen that y^+^ exhibits a higher value near the inlet section than on other surfaces due to the entry effect. However, the entry effect is rather limited before entering the first unit cell. Overall, y^+^ is less than 1.0 on both the lattice core and the facesheets, which demonstrates that the size of the first-layer mesh satisfies the demand of the near-wall function. Thus, the mesh sensitivity and the turbulent model are reliable for conducting the subsequent simulations.

Table 3 first presents a comparison of the temperature distribution between the predicted results and the measured data for the four test cases at the design flow velocity of 15.0 m/s. The temperature variations obtained numerically and experimentally agree reasonably well with each other, and a deviation within 9.2% is exhibited. Specifically, for the temperature measured near the inlet section, excellent prediction with a maximum error of 5.0% is achieved. Table 4 further compares the predicted bottom temperature distribution with the experimental data for Kagome OB at various flow velocities. The present numerical results are also in reasonable agreement with the experimental data, showing a deviation within 9.7%. Hence, the numerical model is believed to be suitable for clarifying the heat transfer characteristics of the sandwich panels.

**Table 3:** Predicted bottom temperature with the experimental data at the design flow velocity of 15.0 m/s.

Test case	N1 (°C)		N2 (°C)		N3 (°C)		N5′ (°C)	
	Numerical	Error	Numerical	Error	Numerical	Error	Numerical	Error
Kagome OA	46.5	1.7%	—	—	79.3	5.3%	65.8	2.3%
Kagome OB	58.8	5.0%	35.9	5.0%	—	—	49.1	9.2%
Tetrahedron OA	50.4	4.5%	—	—	65.9	5.3%	60.2	8.2%
Tetrahedron OB	61.8	6.8%	47.5	1.5%	—	—	58.3	4.3%

**Table 4:** Predicted bottom temperature distribution with the experimental data for Kagome OB.

Flow velocity m/s	N1 (°C)		N2 (°C)		N5′ (°C)	
	Numerical	Error	Numerical	Error	Numerical	Error
5.5	80.1	3.0%	54.2	7.3%	69.7	5.6%
9.4	66.9	4.2%	39.4	4.8%	54.1	9.7%

## Numerical Results and Discussion

### Overall heat transfer performance

The overall heat transfer performance of a lattice core sandwich panel can be described using three dimensionless parameters, the Nusselt number $N{u}_{H}$, the Reynolds number $R{e}_{H}$, and the pressure drop coefficient ${f}_{H}$. Choosing the unit cell height ($h$) as the characteristic length, the three parameters are defined as follows:

2 $${R}{{e}}_{H}=\frac{{\rho }_{f}{U}_{m}h}{{\mu }_{f}},\,N{u}_{H}=\frac{{d}_{p}h}{{k}_{f}},\,{\rm{and}}\,{f}_{H}=x=\frac{{\rm{\Delta }}P}{{\rm{L}}}\frac{h}{{\rho }_{f}{U}_{m}^{2}/2}$$

where ${\rho }_{f}$, ${\mu }_{f}$, and ${k}_{f}$ are the density, the dynamic viscosity, and the thermal conductivity of air, respectively. ${U}_{m}$ is the inlet velocity, and ${\rm{\Delta }}P$ refers to the pressure drop. ${d}_{p}$ is the heat transfer coefficient, defined as:

3 $${d}_{p}=\frac{Q}{{A}_{up{\rm{\Delta }}T}}$$

where ${A}_{up}$ is the area of the heating surface. For the iso-temperature boundary condition, the temperature difference ${\rm{\Delta }}T$ is defined as^36^:

4 $${\rm{\Delta }}{\rm{T}}=\frac{({T}_{w}-{T}_{in})-({T}_{w}-{T}_{out})}{ln[({T}_{w}-{T}_{in})-({T}_{w}-{T}_{out})]}$$

where ${T}_{in}$, ${T}_{out}$, and ${T}_{w}$ are the temperatures at the inlet, the outlet, and the bottom endwall.

**Figure 7:**
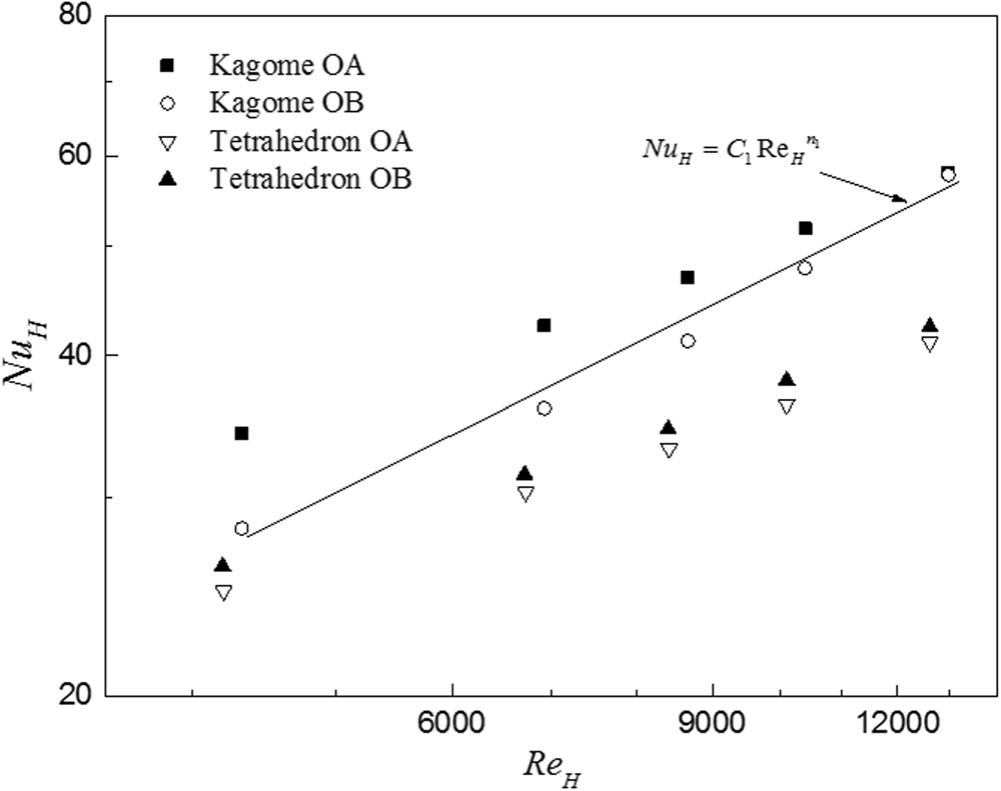
Overall Nusselt number for four test cases as a function of Reynolds number.

Based on the validated DCRU numerical model, the overall heat transfer values for the four test cases are quantified in Fig. 7. With the increment of the Reynolds number, the Nusselt number for all test cases ascend gradually. However, even if the sandwich panels have the same relative density, specific surface area and material thermal conductivity, the heat dissipation performance diverges obviously for the four test cases due to the difference in geometrical morphology and the topological orientation. Overall, the Kagome lattice exhibits excellent heat exchange performance compared to the tetrahedral lattice, especially at high Reynolds number. Among them, Kagome OB exhibits 8~37% higher Nusselt number than tetrahedron OB. For a given geometrical morphology, the heat transfer performance of Kagome OA is higher than that of Kagome OB, while it is similar for two orientations within the tetrahedral lattice. Further, the numerical results found the applied heat flux for Kagome OB is less than that for Kagome OA, which leads to the lower mean temperature for Kagome OB as revealed in the experimental results even if the capacity of heat removal for the latter is more effective than that for the former. It is expected that the divergence of the thermal performance may become more pronounced as the thermal conductivity of the lattice increases.

Correspondingly, the calculated Nusselt number is correlated as a function of the Reynolds number as:

5 $$R{e}_{H}={\rm{C}}R{e}_{H}^{n}$$

where the coefficients of the empirical correlations for the four test cases are listed in Table 5, with all the correlation coefficients greater than 0.98. Particular focus is placed upon the two orientations of Kagome lattice. Although the difference between the two orientations is obvious at low Reynolds number, it can be seen that the slope of Kagome OB is higher than that of Kagome OA. Thus, the effect of topological orientation is negligible beyond a relatively high Reynolds number, consistent with the results of Fig. 7. A similar observation was found by Joo^31^ for WBK lattice in experiment. However, it should be noted that the correlation is only applicable in the present Reynolds number range (4,330 < *Re*_*H*_ < 13,000).

**Table 5:** Empirical correlations for four test cases.

	Kagome OA	Kagome OB	Tetrahedron OA	Tetrahedron OB
*C*	0.4861	0.0833	0.5148	0.6007
*n*	0.5754	0.6882	0.4624	0.4499

### Comparison of fluid flow and heat transfer characteristics

To understand the underlying heat transfer enhancement mechanisms for the two lattice, the detailed flow patterns and their effect on heat transfer characteristics of various surfaces are compared between Kagome OB and tetrahedron OB, which represent the typical geometric morphologies for the two lattice cores.

**Figure 8:**
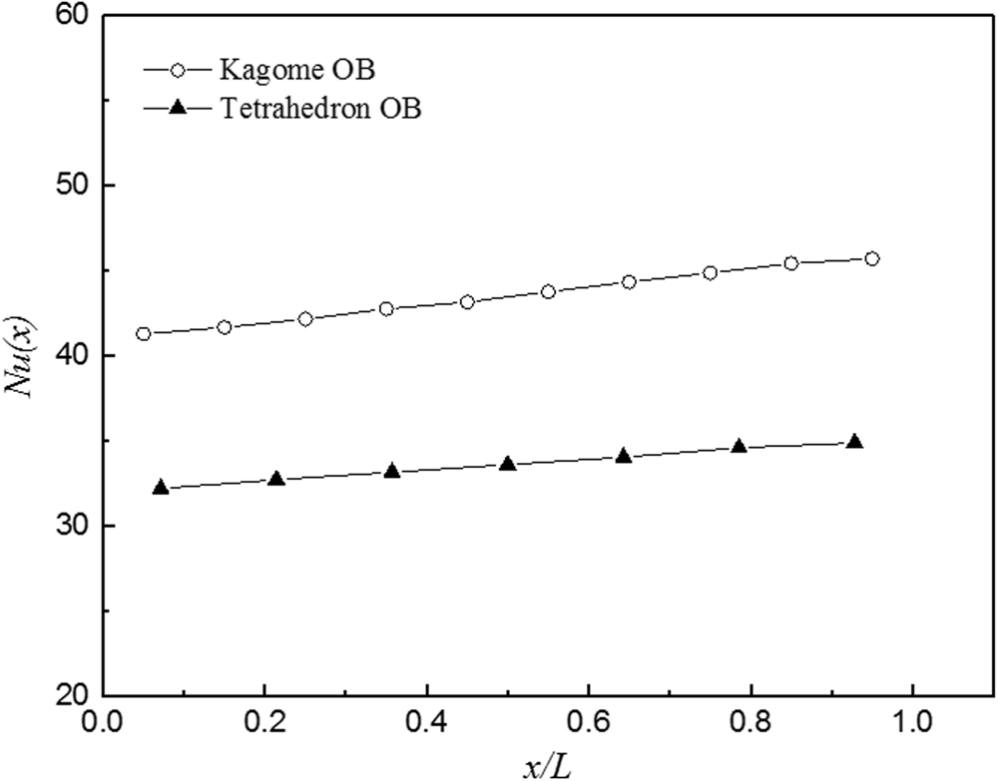
The streamwise variation of Nusselt number at *Re*_*H*_ = 8570.

As the basis of a reliable comparison between Kagome OB and tetrahedron OB, the entry and exit region effects on overall heat transfer have to be classified first to ensure the heat dissipation in approximately fully developed thermal flow^38^. Figure 8 presents the variation of the Nusselt number along the streamwise direction with a Reynolds number of 8,570. For both sandwich panels, the Nusselt number slightly increases from the first unit cell to the last unit cell, which is consistent with the trend observed for X-type lattice^3^. The exit effect is observable within the last unit cell as the trend of ascendance is slow down. However, the increment is limit, with a 10.7% and 8.3% higher Nusselt number in the last unit cell than that in the first unit cell for Kagome OB and tetrahedron OB, respectively. It implies that the flow and thermal boundary layers are developing; therefore, the subsequent comparison for the two sandwich panels is reliable.

**Figure 9:**
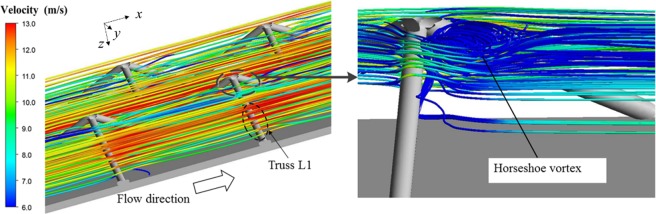
Fluid flow patterns of tetrahedron OB: (**a**) overview of primary flow; (**b**) detail flow feature.

Consequently, Fig. 9(a) presents the fluid flow patterns of tetrahedron OB in terms of streamlines. It is observed that the primary flow of tetrahedron OB is generally straight and parallel to the endwalls, while the lattice core changes the airflow condition and promotes disordered flow locally at the regions of vertices. Hence, the phenomenon of stagnation and separation appears, and the horseshoe vortex forms when the fluid flows around the trusses [Fig. 9(b)]. The large flow resistance near the vertices can cause a high tangential velocity, which leads to the relatively high local heat transfer efficiency.

**Figure 10:**
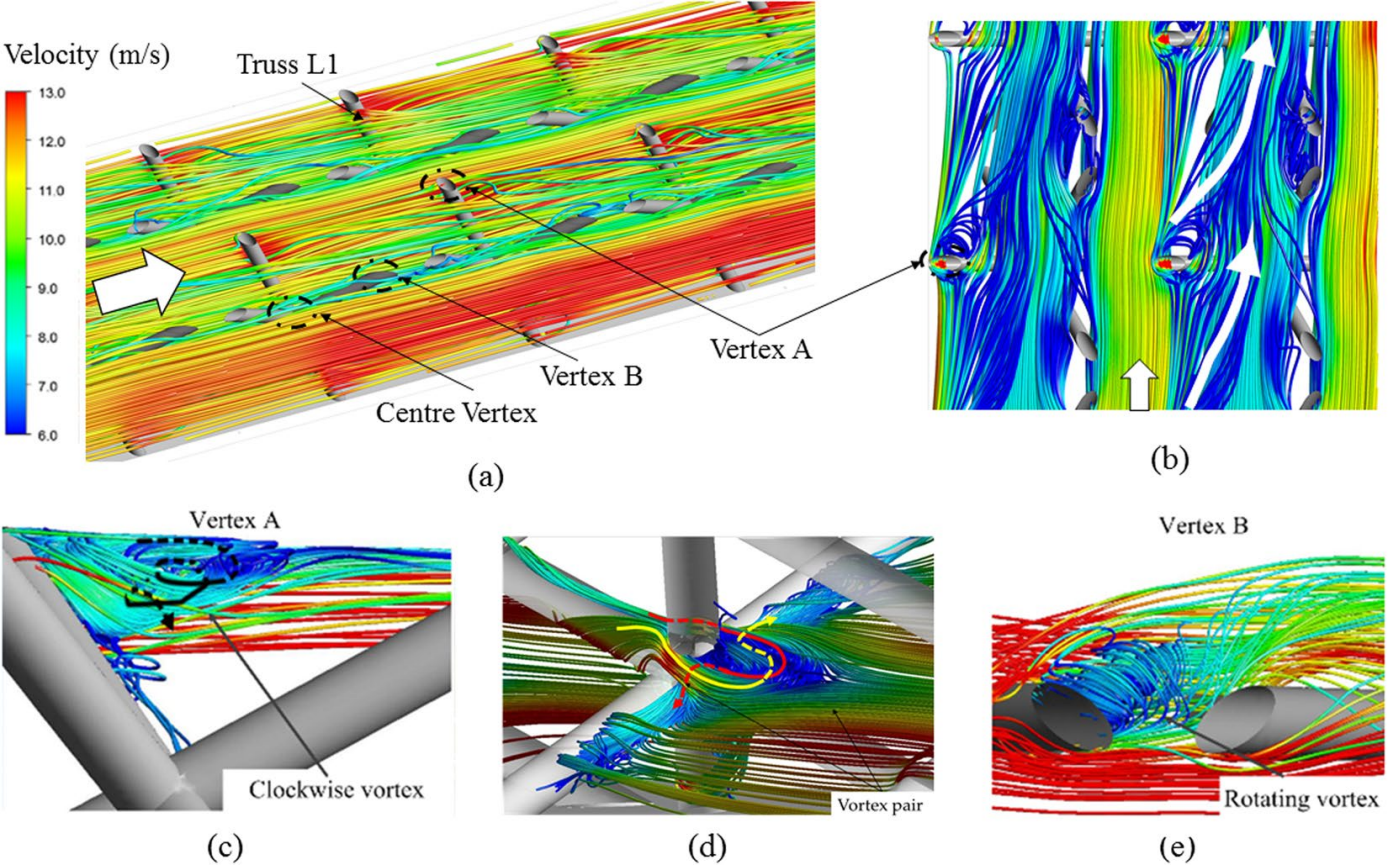
Fluid flow patterns of Kagome OB: (**a**) overview of primary flow; (**b**,**c**) detailed flow features near the vertex A and center vertex, respectively; (**d**) flow features near the vertex B.

However, due to the change in geometrical morphology from tetrahedron OB to Kagome OB, the blockage ratio of flow area by solid trusses is improved, which leads to more irregular primary flow [Fig. 10(a)]. Especially, two types of flow motion appear when the fluid flows passing through the vertex A of truss L1 intersected with the upper endwall. Within the lattice core, a tangential flow caused by the strong shear from the primary flow is induced near the boundary of endwall, as highlighted in Fig. 10(b). It is continually fed by the rear flow and the separated vortex as will be shown in Fig. 10(c); therefore, becomes predominant gradually with the developing flow. Such a tangential flow motion undoubtedly intensifies the transverse flow mixing. Additionally, the large flow separation occurs and a clockwise vortex (viewed from above) is formed behind the vertex A. Under the influence of inclined truss, the vortex becomes skewed and spirals towards the centre vertex. During the process, it mixes with the tangential flow and fades away gradually until it meets with the vortex from the centre vertex.

Meanwhile, there is also a similar flow pattern appearing at the vertex of truss L1 intersected with the bottom endwall (vertex B). However, the additional vertices in the centre of the trusses for Kagome OB act as a robust vortex generator, which is different from the flow pattern of tetrahedron OB. A pair of vortex forms behind the centre vertex of the Kagome cell, as shown in Fig. 10(d). When flowing across the centre vertex, the upstream fluid separates into two secondary flows. One of the secondary flows partially feeds fluid behind the centre vertex clockwise, resulting in one leg of the vortex pair. Similarly, the other secondary flow also feeds fluid behind the same vertex anticlockwise, causing the other leg of the vortex pair. Subsequently, due to the incline of truss L1, the fluid of the vortex pair climbs up or flows down along the truss and mixes with the vortex near the upper or bottom endwalls.

For the other vertices of the Kagome lattice cell, irregular vortical flow patterns also exist behind the vertices of truss L2 and truss L3, which are similar to those in tetrahedron OB. Figure 10(e) shows the formed rotating vortex as the boundary layer flows roll up.

**Figure 11:**
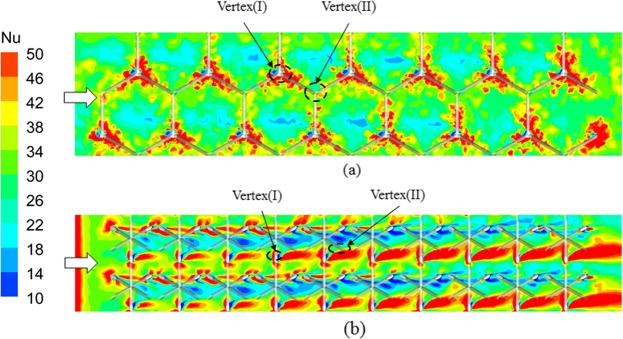
Local heat transfer distribution on upper endwall: (**a**) Kagome OB; (**b**) tetrahedron OB.

The local heat transfer is directly related to the fluid flow feature adjacent to the endwall. Specifically, the vortex induced by the trusses creates the region of flow recirculation and reattachment, which can greatly improve the local heat transfer. Figure 11 compares the heat transfer distributions on the bottom endwalls of tetrahedron OB and Kagome OB in terms of the local Nusselt number, which is calculated based on the temperature difference defined in Eq. (4). Note that the vertex (I) and vertex (II) are joints of trusses intersected with the bottom endwall and the upper endwall, respectively. Corresponding to the result shown in Fig. 8, the local Nusselt number increases along the streamwise direction due to the gradual intensified flow mixing. The entry region effect is rather limited in the first unit cell for both of the sandwich panels. Overall, the local heat exchange near the vertical regions is enhanced corresponding to the complex flow separation and stagnation. For tetrahedron OB, the high heat transfer region is only clearly visible around vertex (I) due to the horseshoe vortex revealed in Fig. 9(b). However, the regions of evidently high Nusselt number for Kagome OB are observable not only around vertex (I) but also along the downstream of each truss. The enhancement is mainly attributed the fact that the tangential flow shown in Fig. 10(b) as well as the rotating vortex [Fig. 10(d)] facilitates to the heat exchange between the endwalls of sandwich panel and cooling air as a result of strong shear to the endwall. For quantitative evaluation, the average Nusselt number of Kagome OB on the endwalls is 31% higher than that of tetrahedron OB, which indicates one contributor to the enhancement of heat exchange performance of Kagome OB.

**Figure 12:**
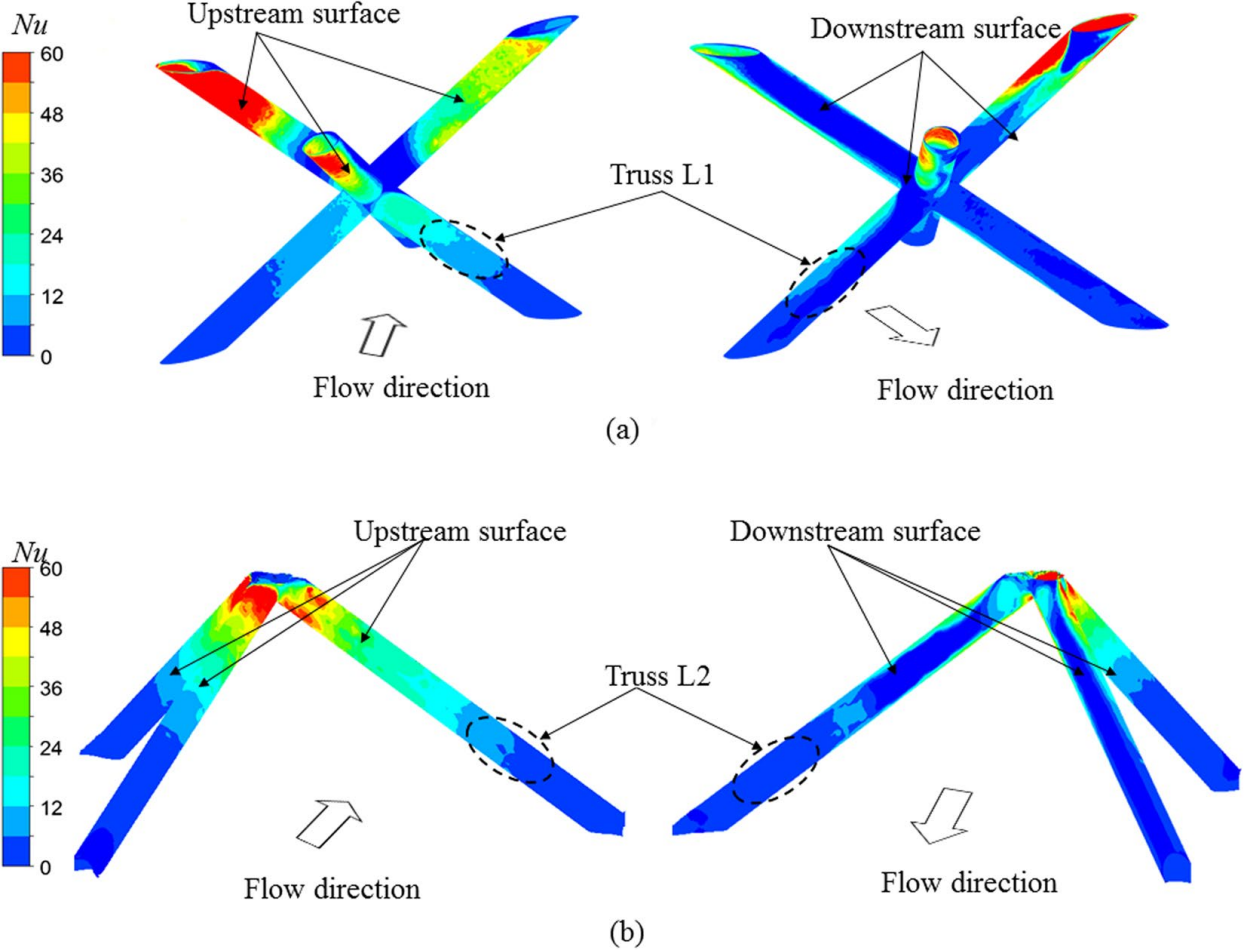
Local heat transfer distribution on lattice core: (**a**) Kagome OB viewed from the upstream and downstream, respectively; (**b**) tetrahedron OB viewed from the upstream and downstream, respectively.

In addition to enhancing the endwall heat transfer, the vortical flow also promotes heat transfer performance on the trusses of Kagome and tetrahedral lattices. Figure 12 compares the heat transfer characteristics of the lattice core in Kagome and tetrahedral unit cells. Note that truss L2 for both lattice cores is perpendicular to the flow direction, the surfaces of the trusses can be divided into two different types: the upstream surface and the downstream surface. Local heat transfer is dominated by the fluid flow impinging onto the edge regions, which leads to a higher local Nusselt number than that on the downstream surface. However, compared with tetrahedron OB, Kagome OA exhibits an evidently higher Nusselt number on the upstream surface, as it has more joints connected to the endwall, promoting the heat exchange between the trusses and the cooling air. It clearly enhances the local heat transfer relative to tetrahedron OB. Additionally, the heat transfer enhancement for Kagome OB also occurs within the region around the centre vertex, which acts as the robust vortex generator shown in Fig. 10(c). Therefore, the lattice core in Kagome OB provides an approximately 14% higher average Nusselt number than that in tetrahedron OB. Meanwhile, the lattice core in Kagome OB has a 45% higher surface area relative to tetrahedron OB at a given porosity, which is also responsible for the heat transfer enhancement mechanism.

Table 6 summarizes the overall heat transfer mechanisms, with the contribution of each surface evaluated as the product of the average Nusselt number and the heat transfer area. In general, the endwall devotes more than four-fifths of overall heat removal to the sandwich panels due to the high porosity, 0.985 of the truss-core lattice. For each sandwich panel, it can be found that the lattice core of Kagome OB has an 9% higher contribution compared to that of tetrahedron OB. The higher average Nusselt number and surface area revealed in Fig. 12(b) are the main mechanisms for enhanced heat transfer.

**Table 6:** Contribution of endwall and lattice core to overall heat transfer.

Test case	Endwall	Lattice core
Tetrahedron OB	89%	11%
Kagome OB	80%	20%

#### Pressure drop

**Figure 13:**
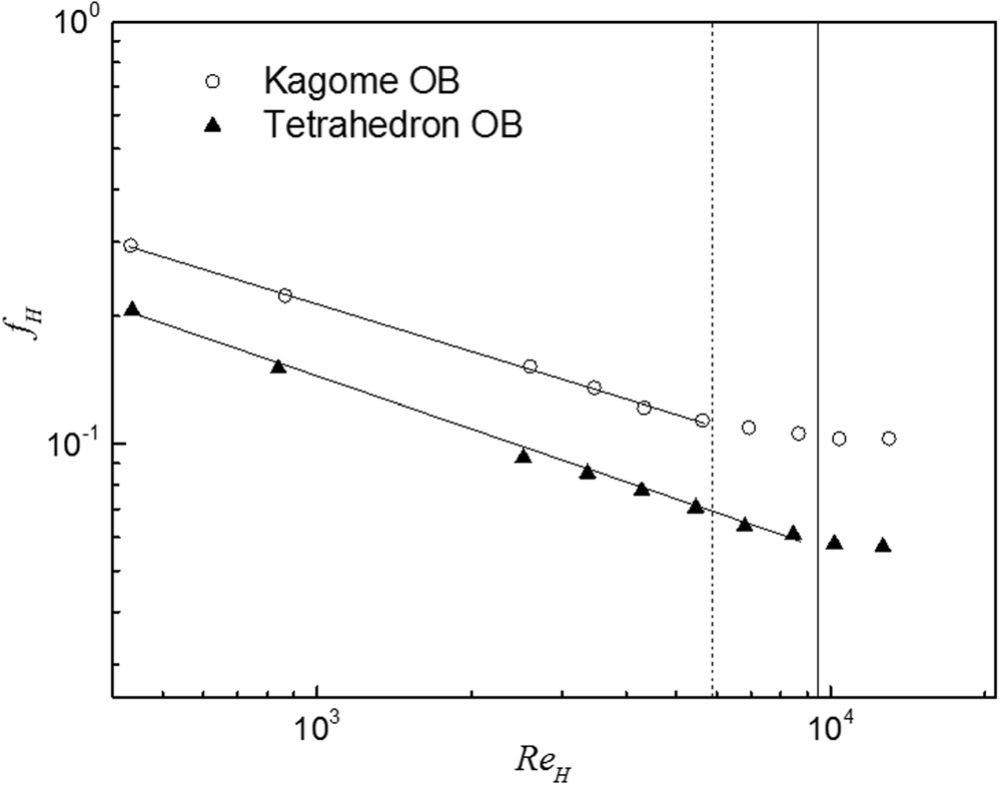
The friction factor for the two sandwich panels as a function of Reynolds number.

Pressure drop characteristics evaluated in terms of the friction factor defined in Eq. (4) for Kagome OB and tetrahedron OB are shown in Fig. 13. For Kagome OB, the flow is laminar when *Re*_*H*_ < 5800, and it is in transition from the laminar regime to the turbulent regime when 5800 < *Re*_*H*_ < 9500. The flow becomes turbulent when *Re*_*H*_ > 9500, with an approximately constant friction factor of 0.103. Kagome OB presents a similar friction factor trend while the flow transitions to the turbulent regime until *Re*_*H*_ > 8500. It finally keeps an approximately constant value of 0.057. Therefore, Kagome OB causes nearly twice the pressure drop that tetrahedron OB does. The complex flow mixing reduced by the arrangement of the lattice truss core is believed to be the main mechanism for the higher pressure drop. More pumping power would be needed for the thermal protection system for Kagome OB than for tetrahedron OB.

## Conclusions

This paper performed comparisons of the thermal insulation, the inner flow pattern and the Nusselt number distribution of the sandwich panels with the Kagome lattice core and the tetrahedral lattice core in the forced convective heat transfer condition. The thermal insulation (in terms of the temperature difference between the heating surface and the cooling surface) was obtained from the forced convective heat transfer experiments, while the inner flow pattern and the Nusselt number distribution were captured by numerical simulation using CFX 17.0.

The tested sandwich panels for forced convective heat transfer experiments were manufactured at the same density from titanium alloy TC4 by 3D printing technology. Four tests were carried out by using the two specimens and forcing the cooling air to flow along two perpendicular orientations OA and OB. From the measured temperature of the cooling surface, it can be concluded that the sandwich panel cored with the Kagome lattice achieves the highest thermal insulation when the air flows in the orientation OB under the identical temperature loadings.

The cooling airflow pattern within the Kagome lattice is more irregular than that within the tetrahedral lattice. In the Kagome lattice core, the additional centre vertex disturbs the primary flow and induces a pair of vortices formed behind the centre vertex. Intensified flow stagnation and separation cause high tangential velocity vorticity near the vertices, which is the underlying mechanism for heat transfer enhancement.

The complex fluid flow behaviours in the Kagome lattice enhance heat transfer on both the endwalls and the trusses. For a given Reynolds number, the average Nusselt number on the endwalls of the Kagome lattice sandwich panel is 31% higher than that of the tetrahedral lattice sandwich panel, with the air flowing along the direction OB. Furthermore, the average Nusselt number of the Kagome lattice core is 14% higher than that of the tetrahedral lattice core. In view of these heat transfer enhancements, the sandwich panel cored with the Kagome lattice provides an 8~37% higher overall Nusselt number compared to that cored with the tetrahedral lattice within the Reynolds number range of interest (4,330–13,000).

For a given Reynolds number, the Kagome lattice core causes approximately twice the pressure drop that the tetrahedral lattice core does, which indicates that a higher pumping power would be needed for cooling the sandwich panel with the Kagome lattice core than for that with the tetrahedral lattice core.

## Data Availability

The datasets generated during and/or analyzed during the current study are available from the corresponding author on reasonable request.
